# Squeeze Film Effect in Surface Micromachined Nano Ultrasonic Sensor for Different Diaphragm Displacement Profiles

**DOI:** 10.3390/s23104665

**Published:** 2023-05-11

**Authors:** Avik Ghosh Dastidar, Reshmi Maity, Ramesh Chandra Tiwari, Dejan Vidojevic, Tijana S. Kevkic, Vojkan Nikolic, Subhajit Das, Niladri Pratap Maity

**Affiliations:** 1 Regent Education and Research Foundation Group of Institutions, Kolkata 700121, India; avikgd@regent.ac.in; 2 Department of Physics, Mizoram University (A Central University), Aizawl 796004, India; rctiwari@mzu.edu.in; 3 Department of Electronics and Communication Engineering, Mizoram University (A Central University), Aizawl 796004, India; mzut138@mzu.edu.in (R.M.); mzut133@mzu.edu.in (N.P.M.); 4 Department of Information Technology, University of Criminal Investigation and Police Studies, 11060 Belgrade, Serbia; vojkan.nikolic@kpu.edu.rs; 5 Department of Physics, Faculty of Sciences and Mathematics, University of Pristina in Kosovska Mitrovica, 38220 Kosovska Mitrovica, Serbia; tijana.kevkic@pr.ac.rs; 6 Functional Materials and Devices Division, CSIR-Central Glass and Ceramic Research Institute, Kolkata 700032, India; subhajit@cgcri.res.in

**Keywords:** CMUT, Reynold’s equation, displacement profile, pressure profile, modified Bessel’s equation, FEM

## Abstract

In the present paper, we have analytically explored the small variations of the local pressure in the trapped air film of both sides of the clamped circular capacitive micromachined ultrasonic transducer (CMUT), which consists of a thin movable membrane of silicon nitride (Si_3_N_4_). This time-independent pressure profile has been investigated thoroughly by solving the associated linear Reynold’s equation in the framework of three analytical models, viz. membrane model, plate model, and non-local plate model. The solution involves Bessel functions of the first kind. The Landau–Lifschitz fringing technique has been assimilated to engrave the edge effects in estimation of the capacitance of CMUT, which should be considered in the micrometer or lesser dimension. To divulge the dimension-based efficacy of the considered analytical models, various statistical methods have been employed. Our use of contour plots of absolute quadratic deviation revealed a very satisfactory solution in this direction. Though the analytical expression of the pressure profile is very cumbersome in various models, the analysis of these outputs exhibits that the pressure profile follows the displacement profile in all the cases indicating no viscous damping. A finite element model (FEM) has been used to validate the systematic analyses of displacement profiles for several radii and thicknesses of the CMUT’s diaphragm. The FEM result is further corroborated by published experimental results bearing excellent outcome.

## 1. Introduction

Nowadays, pressure sensors play an important role in different fields of applications [1,2,3]. Conventional bulk-piezo transducer is a common pressure sensor, though it has some disadvantages over CMUT, such as geometry constraint for choice of frequency, failure in high temperatures, and non-preference for operation in air (due to high impedance mismatch between piezoelectric material and air) [4]. On the other hand, the cost-effective applications of CMUT in the field of non-destructive testing and evaluation (NDE) [5,6,7,8,9], have the precedence over piezoelectric transducers in different aspects (e.g., superior bandwidth, facile batch fabrication, better interfacing capability, suitability for air-coupled non-destructive applications) [10,11], and thus inspires the investigation of its appropriate design before fabrication.

In the year 2004, Yongli Huang et al. have carried out a comparative study of the performance of CMUT for three different membrane configurations [12]. A compact analytical model was proposed by R. Maity et al. to calculate the dependency of collapse voltage on different physical parameters and structural features of the device [13,14]. In another study, the authors have used Mason’s equation to establish a circular membrane model [15]. Mohammad Maadi et al. have developed a non-linear large-signal equivalent model of square CMUT, showing a good agreement with the FEM result [16]. Another recent study performed by M. Pal et al. has focused on the electrical capacitance and electrostatic force on the membrane of CMUT [17]. Employing the well-established plate theory by Von Karman and the single-mode Galerkin decomposition method, M Saadatmand et al. have proposed one and two-sided CMUT systems [18]. Moreover, there are several studies regarding the pressure profile inside the CMUT [19,20,21,22,23,24,25]. Zand and Ahmadian [19,20] have studied the phenomena, such as vibrational behavior and dynamics of multi-layer microplates, using the coupled first-order shear deformation theory (FSDT) and finite difference method (FDM). Furthermore, the authors have suggested an approach to investigate the dynamics of microplates when squeeze film damping and non-linear electrostatic force are engaged. Casimir force and squeeze film damping have been addressed by Dastani et al. [21] in relation to the pull-in instability and dynamic behavior of electrostatically actuated nano- and micro-electro mechanical systems (MEMS). Hosseini et al. [22] have studied the dynamic pull-in and snap-through behavior for curved beams with micro/nano dimensions, considering the squeeze film damping effect. In 2017, Abderezaei and Zand [20] have modeled the dynamics of the microbeams under the effects of electrostatic force, mechanical shock, squeeze film damping (SQFD), and fringing field using a Galerkin-based reduced-order model. Lotfi et al. [21] have modeled and validated the transient behavior and dynamic pull-in instability of electrostatically-actuated fluid-conveying micro-bridges and micro-cantilevers. Roozbahani et al. [22] have studied the dynamic behavior of a micro arch-shaped beam under different loading conditions, including electrostatic force, axial force, and mechanical shock loading in the presence of squeeze film damping.

Employing arbitrary acoustic venting circumstances, the theoretical research of Darling et al. [26] have produced an analytical model for the forces that come from compressible squeeze film damping. Ahmad et al. [27] have explained that in the sealed cavities, the reaction force due to the air trapped inside the cavity is completely a pure spring force, with the displacement in the phase for all frequencies having no viscous damping, even if the flexure of the plate is taken into account. Apte et al. [28] have fabricated CMUTs with vented cavities with plate radii ranging from 750 μm to 1500 μm. Primarily, they can be efficiently used for flare gas metering. Moreover, the authors have observed two resonance frequencies from the vented CMUTs and explained them as the mechanical resonance of the plate and an acoustic Helmholtz resonance associated with the cavity and the venting vias. Younis et al. [29] have studied the squeeze film damping of microplates actuated by large electrostatic loads.

Inspired by these existing scientific explanations, in the present work, we have considered the linear Reynold’s equation and analytically determined its time-independent solution, which is the most appropriate theory in our case of finding small variations of the local pressure in the trapped air of both sides of clamped circular CMUT, which consists of a thin movable membrane of Si_3_N_4_. The mechanical properties, as well as the static and dynamic aging tests [30,31], have proven the potential of silicon nitride as a structural material in MEMS. In our study, to increase the sensitivity of detection of small changes in local pressure, the large membrane dimension and high electrostatic load are preferred. A silicon carbide-based structure of CMUT was modeled and studied by M. Pal et al. [32]. has been carried out. To solve the Reynold’s equation, we have used the separation of variables method, taking into account the flexural motion of the top vibrating plate. To systematically study the pressure profile, the displacement of the membrane should be given accurately. For this purpose, we have considered three different theoretical models, termed as the membrane model [33], plate model [34,35], and non-local plate model [36]. The Landau-Lifschitz model for capacitance fringing has been embraced explicitly in all of the three methods to accurately calculate the capacitance of the CMUT [37]. We have also made an effort to determine which theoretical model is preferred when determining the displacement profile based on the physical dimension of the CMUT. For this purpose, we have carefully examined the CMUTs by changing dimensions, and the analytical results are then censoriously analogized with the FEM outputs of COMSOL using various relevant statistical methods. The study of contour plots of absolute quadratic deviation (QD), between the displacement values of model and FEM, revealed very interesting observations consistently regarding the performance of these models.

## 2. Analytical Model

**Figure 1:**
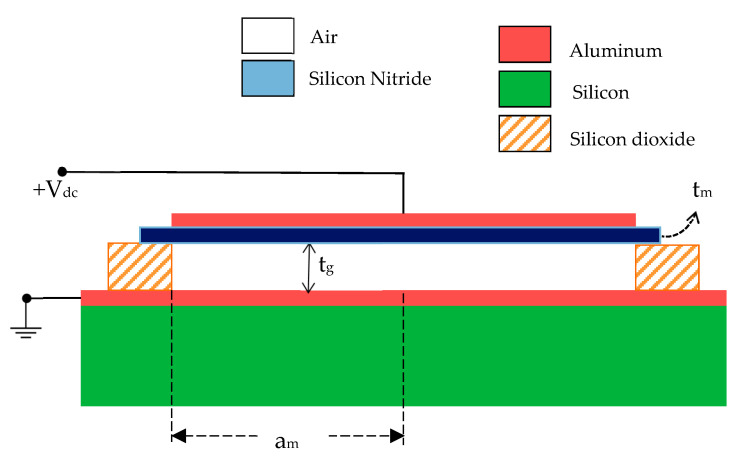
Structure of Si_3_N_4_ CMUT with membrane thickness $t_{m}$ and air gap thickness $t_{g}$.

Figure 1 depicts the two-dimensional structure of the CMUT that was taken into consideration for this investigation. It consists of an aluminum (Al) top electrode on a thin movable membrane of Si_3_N_4_. The properties, such as low density, high resistance for thermal shock, wear and oxidation, along with the substantial fracture toughness and temperature strength, inspired us to choose Si_3_N_4_ as the vibrating membrane material of the transducer. The membrane is placed above a heavily doped silicon substrate separated by a small air gap forming a capacitor. The layer of metallic Al is deposited over this substrate to act as the ground electrode. The wall of the air gap is made up of the insulator SiO_2_.

When a DC bias is applied between the top and bottom electrodes of the device, an attractive electrostatic force is developed between the electrodes of the capacitor, which in turn, causes a deflection of the membrane toward the fixed bottom electrode. This deflection is resisted by the stiffness of the membrane, causing equilibrium. When an AC voltage with an ultrasonic frequency range is applied over the DC bias, the membrane vibrates with the same frequency and produces ultrasound.

For a small capacitor, the edge effect cannot be disregarded. Therefore, the fringing effect must be explicitly included. The total capacitance of this small-scale capacitor, such as CMUT, is the sum of the conventional and fringing capacitances, i.e., $C_{Total}=C_{Conventional}+C_{Fringe}$. The capacitance at the radial position r of a circular plate capacitor with radius a, thickness t, and permittivity $\left(\varepsilon \right)$ can be stated, in general, as taking into account the Landau-Lifschitz method [34] for fringing.

(1) $$C=\left(\frac{\varepsilon \pi a^{2}}{t}\right)+\varepsilon r\ln\left(\frac{16\pi a}{t}-1\right)$$

In the presently considered CMUT, there are two circular capacitors, viz. membrane capacitor (with capacitance $C_{mf}$) and air gap capacitor (with capacitance $C_{gf}$) in series. The capacitance of each of them should be calculated as suggested in the general Equation (1). After considering fringing correction, the overall capacitance of the CMUT is $C_{eqf}$, which is given by Equation (2):

(2) $$C_{eqf}=\frac{C_{mf}C_{gf}}{C_{gf}+C_{mf}}$$

If a DC bias $V_{dc}$ across the capacitance, $C_{eqf}$, is applied, its stored potential energy U is written as follows:

(3) $$U=\frac{1}{2}\times \left(C_{eqf}\times V_{dc}^{2}\right)$$

The electrostatic force that results from this can be written as follows:

(4) $$F_{e}=-\frac{dU}{dt_{g}}=-\frac{1}{2}\times V_{dc}^{2}\times \left(\frac{dC_{eqf}}{dt_{g}}\right)$$

Due to the direct interrelation between the change in capacitance and the variation in air gap thickness $\left(t_{g}\right)$, fringing-induced capacitance enhancement causes a considerable rise in electrostatic force. The membrane’s and the air gap’s combined fringing capacitances describe the entire electrostatic force acting on the CMUT as follows:

(5) $$F_{e}=\frac{V_{dc}^{2}{C_{mf}}^{2}}{2{C_{gf}}^{2}{\left(\frac{C_{mf}}{C_{gf}}+1\right)}^{2}}\left[\varepsilon_{g}\left(\frac{\pi {a_{m}}^{2}}{{t_{g}}^{2}}\right)+\frac{\varepsilon_{g}a_{m}}{\left(\frac{16\pi a_{m}}{t_{g}}-1\right)}\times 16\left(\frac{\pi a_{m}}{{t_{g}}^{2}}\right)\right]$$

Here, $a_{m}$ is the radius of the Si_3_N_4_ membrane and $\varepsilon_{g}$ is the permittivity of squeezed air film.

### 2.1. Pressure Profile

To theoretically evaluate the pressure inside the squeezed air film of the Si_3_N_4_ CMUT, we begin with the Reynold’s equation which is derived from the Navier–Stokes equation. For a fluid film with local pressure P and density ρ, the Reynold’s equation is given by the following:

(6) $$\vec{\nabla }\cdot \left[\frac{\rho W^{3}}{\mu }\vec{\nabla }P\right]=12\frac{\partial \left(\rho W\right)}{\partial t}+6\vec{\nabla }\left(\rho W\bar{U}\right)$$

where μ is the viscosity of the liquid or gas, W is the height of the film at any point of time t, and $\bar{U}$ is the lateral velocity of the moving plate. In this specified problem, lateral velocity $\bar{U}=0$ results in the following:

(7) $$\vec{\nabla }\cdot \left[\frac{\rho W^{3}}{\mu }\vec{\nabla }P\right]=12\frac{\partial \left(\rho W\right)}{\partial t}$$

Considering Boyle’s law for the isothermal expansion of the fluids, such as air, the above equation is transformed into the following:

(8) $$\nabla^{2}P=\gamma^{2}\left(\frac{\partial P}{\partial t}\right)-\gamma^{2}\left(\frac{\partial W}{\partial t}\right)$$

where $\gamma^{2}=\left\{\left(12\mu \right)/\left(w_{0}^{2}P_{a}\right)\right\}$ is a constant term with $w_{0}$ being normal air gap spacing and $P_{a}$ is Ψ ambient pressure. As the present problem has cylindrical symmetry, we use cylindrical coordinate, in which,

$$\nabla^{2}\equiv \frac{\partial^{2}}{\partial r^{2}}+\frac{1}{r}\left(\frac{\partial }{\partial r}\right)+\frac{1}{r^{2}}\left(\frac{\partial^{2}}{\partial \theta^{2}}\right)+\frac{\partial^{2}}{\partial z^{2}}$$

Using two new scaled variables $R=r/a_{m}$, T=ωt, and realizing that the pressure is invariant with respect to the spherical angle θ and varies negligibly with z, Equation (8) is written as follows:

(9) $$\frac{\partial^{2}P}{\partial R^{2}}+\frac{1}{R}\left[\frac{\partial P}{\partial R}\right]=\sigma \left[\frac{\partial P}{\partial T}-\frac{\partial W}{\partial T}\right]$$

where *ω* is the vibration frequency and $\sigma =\gamma^{2}{a_{m}}^{2}\omega$ is the dimensionless squeezed number.

Equation (9) can be solved by using the separation of variable method. Let $P=\Psi \left(R\right)\Phi \left(T\right)$, where Ψ and Φ are the function of R and T, respectively. Now, replacing the values of ∂P/∂R, $\partial^{2}P/\partial R^{2}$, ∂P/∂T, and ∂W/∂T into Equation (9), we have the following:

(10) $$\frac{1}{\left(\Psi -W\right)}\left[\frac{\partial^{2}\Psi }{\partial R^{2}}+\frac{1}{R}\left(\frac{\partial \Psi }{\partial R}\right)\right]=\sigma \left[\frac{1}{\Phi }\left(\frac{\partial \Phi }{\partial T}\right)\right]=-m^{2}$$

Here, m is an arbitrary constant. 

To solve the equation related to Φ, that is $\sigma \left[\frac{1}{\Phi }\left(\frac{d\Phi }{dT}\right)\right]=-m^{2}$ and considering the motion of the membrane to be harmonic with T, we can write the following:

(11) $$\Phi =\Phi_{0}e^{jT}$$

where $\Phi_{0}$ is integration constant and $-m^{2}=j\sigma$. From Equation (10) and considering $-m^{2}=j\sigma$, we can also write the following:

(12) $$R^{2}\left(\frac{\partial^{2}\Psi }{\partial R^{2}}\right)+R\left(\frac{\partial \Psi }{\partial R}\right)-j\sigma \Psi R^{2}=-j\sigma WR^{2}$$

The solution of Equation (12) will give the pressure distribution inside the squeezed air film. The homogeneous part of Equation (12), i.e.,

$R^{2}\left(\frac{\partial^{2}\Psi }{\partial R^{2}}\right)+R\left(\frac{\partial \Psi }{\partial R}\right)-j\sigma \Psi R^{2}=0$ is the modified Bessel’s equation of the form, $x^{2}\left(\frac{\partial^{2}y}{\partial x^{2}}\right)+x\left(\frac{\partial y}{\partial x}\right)-\left(\beta^{2}x^{2}+n^{2}\right)y=0$, where β is a constant. The general solution is given by $y=AI_{n}\left(\beta x\right)+BK_{n}\left(\beta x\right)$. Let us consider $\beta^{2}=i=\sqrt{-1}$, then, $I_{n}\left(x\right)=i^{-n}J_{n}\left(ix\right)=J_{n}\left(i^{3/2}x\right)$. In this problem, order number n=0 results in $I_{0}\left(x\right)=J_{0}\left(ix\right)$. Moreover, the modified Bessel function of the second kind $K_{n}\left(\beta x\right)$ tends to be an infinity as the argument approaches zero, and thus, is discarded from the solution. Therefore, the following equation can be written:

(13) $$\Psi \left(R\right)=C_{1}J_{0}\left(\sqrt{-j\sigma }R\right)$$

where $C_{1}$ is a constant. To obtain the particular solution, we need the expression for the local gap, or specifically, the Si_3_N_4_ membrane displacement W that we shall evaluate in the following sections employing three models, namely, membrane model, plate model, and non-local plate model; thereby, the total solution for Ψ will be obtained. This study considers a cylindrical-shaped squeezed air film CMUT with z-symmetry and azimuthal symmetry.

### 2.2. Membrane Model

While being actuated by a DC bias $V_{dc}$ in the membrane model, which considers Mason’s analysis, the circular diaphragm’s displacement profile is given by the following:

(14) $$r^{2}\left(\frac{d^{2}W_{m}}{dr^{2}}\right)+r\left(\frac{dW_{m}}{dr}\right)=-\left(\frac{P_{dc}}{T_{m}}\right)r^{2}$$

where r is the radial position of the circular membrane’s surface area $A_{m}=\pi a_{m}^{2}$, $P_{dc}=F_{e}/A_{m}$ is electrostatic pressure, $W_{m}$ indicates membrane displacement, membrane tension is $T_{m}=R_{S}\times t_{m}$, residual stress is $R_{S}$, and $t_{m}$ is the Si_3_N_4_ membrane thickness.

The electrostatic force $F_{e}$ is calculated in accordance with Equation (5). The differential Equation (14) has been solved to achieve the membrane displacement of CMUT and can be expressed as follows:

(15) $$W_{m}=\left(\frac{P_{dc}}{4T_{m}}\right)\left(a_{m}^{2}-r^{2}\right)$$

while solving Equation (12) for the membrane model, we can consider $W=W_{m}$ as given in Equation (15). To obtain the particular solution of (12), let

(16) $$\Psi \left(R\right)=a_{1}R^{2}+a_{2}R+a_{3}$$

Placing the values of Ψ, $\Psi^{\prime }$, and $\Psi^{\prime \prime }$ into Equation (12), and comparing the coefficients of various powers of R on both sides, we can determine the constants as $a_{1}=-W_{m}^{\prime }$, $a_{2}=0$, and $a_{3}=-\left\{\left(W_{m}^{\prime }(4-j\sigma )\right)/j\sigma \right\}$. Here, $W_{m}^{\prime }=W_{m}\left(r=a_{m}\right)$. Using the values of $a_{1}$, $a_{2}$, $a_{3}$ in Equation (16), we achieve, $\Psi \left(R\right)=-W_{m}^{\prime }\left[\left(R^{2}-1\right)+\left(4/j\sigma \right)\right]$. Therefore, the total solution is as follows:

(17) $$\Psi (R)=C_{1}J_{0}\left(\sqrt{-j\sigma }R\right)-W_{m}^{\prime }\left[\left(R^{2}-1\right)+\left(\frac{4}{j\sigma }\right)\right]$$

The $C_{1}$ is evaluated by applying the boundary condition dΨ/dR=0 at R=1, and the resulting solution in the case of membrane model is as follows:

(18) $$\Psi \left(R\right)=\frac{-2W_{m}^{\prime }}{\left(\sqrt{-j\sigma }\right)J_{1}\left(\sqrt{-j\sigma }\right)}J_{0}\left(R\sqrt{-j\sigma }\right)-{W^{\prime }}_{m}\left[\left(R^{2}-1\right)+\left(\frac{4}{j\sigma }\right)\right]$$

### 2.3. Plate Model

In the plate model [16], the displacement $W_{p}$ at radial position r of a CMUT due to the application of electrostatic pressure $P_{dc}$ is written as follows:

(19) $$\frac{d}{dr}\left[\frac{1}{r}\frac{d}{dr}\left(r\frac{dW_{p}}{dr}\right)\right]=\frac{P_{dc}r}{2D}$$

where flexural rigidity $D=\left\{\left(E{t_{m}}^{3}\right)/\left\{12\left(1-\nu^{2}\right)\right\}\right\}$, E is the Young’s modulus, and ν is the Poisson’s ratio. Integrating the above equation, the displacement can be represented as follows:

(20) $$W_{p}=\frac{P_{dc}r^{4}}{64D}+C_{2}\left(\frac{r^{2}}{4}\right)+C_{3}\left(\log\frac{r}{a_{m}}\right)+C_{4}$$

To evaluate the integration constants $\left(C_{2},C_{3},C_{4}\right)$, boundary conditions are applied, such as the fact that the slope of displacement is zero at radial distance r=0 and $r=a_{m}$. Therefore, the deflection of the circular diaphragm is written as follows:

(21) $$W_{p}=\frac{P_{dc}}{64D}{\left({a_{m}}^{2}-r^{2}\right)}^{2}$$

The natural frequency of the plate of density ρ is calculated using the following formula [23]:

(22) $$f=0.47\times \left(\frac{t_{m}}{a_{m}^{2}}\right)\sqrt{\frac{E}{\rho \left(1-\nu^{2}\right)}}$$

Now, we consider the solution of Ψ using $W=W_{P}$. In the plate model, W of Equation (12) is given by $W_{P}$ of Equation (21). To obtain the particular solution, let,

(23) $$\Psi \left(R\right)=a_{1}R^{4}+a_{2}R^{3}+a_{3}R^{2}+a_{4}R+a_{5}$$

Using the similar techniques previously mentioned, the constants are evaluated as follows: $a_{1}=W_{p}^{\prime }$, $a_{2}=0$, $a_{4}=0$, $a_{3}=\left(16W_{p}^{\prime }-2j\sigma W_{p}^{\prime }\right)/j\sigma$, and $a_{5}=W_{p}^{\prime }+\left\{\left(8j\sigma W_{p}^{\prime }-64W_{p}^{\prime }\right)/\sigma^{2}\right\}$. Here, $W_{p}^{\prime }=W_{p}\left(r=a_{m}\right)$. Using the values of $a_{1}$, $a_{2}$, $a_{3}$, $a_{4}$, $a_{5}$*,* we have evaluated the total solution as follows:

(24) $$\begin{array}{l} \Psi \left(R\right)=C_{5}J_{0}\left(\sqrt{-j\sigma }R\right)-\\ W_{p}^{\prime }\left[\frac{64-\sigma^{2}{\left(R-1\right)}^{2}{\left(R+1\right)}^{2}+\left(16R^{2}-8\right)j\sigma }{\sigma^{2}}\right] \end{array}$$

To find $C_{1}$, the value of $C_{5}$, we use the boundary condition at R=1, $\frac{d\psi }{dR}=0$. Now, $J_{n}^{\prime }(x)=\left(n/x\right)J_{n}(x)-J_{n+1}(x)$. Therefore, $J_{0}^{\prime }\left(x\right)=-J_{1}\left(x\right)$ and $J_{0}^{\prime }\left(R\sqrt{-j\sigma }\right)=-J_{1}\left(R\sqrt{-j\sigma }\right)$, and the value of $C_{5}$ can be determined as shown below:

(25) $$C_{5}=\{\left(32W_{p}^{\prime }\right)/\left\{j\sigma \left(\sqrt{-j\sigma }\right)J_{1}\left(\sqrt{-j\sigma }\right)\right\}\}$$

Therefore, the pressure profile inside the CMUT in plate model is written as follows:

(26) $$\begin{array}{l} \Psi \left(R\right)=\frac{32W_{p}^{\prime }}{j\sigma \left(\sqrt{-j\sigma }\right)J_{1}\left(\sqrt{-j\sigma }\right)}\left\{J_{0}\left(R\sqrt{-j\sigma }\right)\right\}-\\ W_{p}^{\prime }\left[\frac{64-\sigma^{2}{\left(R-1\right)}^{2}{\left(R+1\right)}^{2}+\left(16R^{2}-8\right)j\sigma }{\sigma^{2}}\right] \end{array}$$

### 2.4. Non-Local Plate Model

Small scale effects should be carefully considered while studying NEMS devices, and to perform this, the non-local elasticity theory of Morse–Eringen [38,39,40] is more suitable for characterizing the MUT. At equilibrium, equations for a circular plate are constituted as follows:

(27) $$\frac{d}{dr}\left(rQ_{r}\right)=rF_{e}$$

(28) $$rQ_{r}=-\frac{d}{dr}\left(rM_{rr}\right)+M_{\theta \theta }$$

Here, $M_{rr}$ is the radial moment, $Q_{r}$ is the transverse shear force, $M_{\theta \theta }$ denotes the circumferential moment, and $F_{e}$ is the uniformly distributed load, respectively. From [41,42,43,44], the constitutive relations are revealed as follows:

(29) $$\sigma_{rr}-{\left(e_{0}a\right)}^{2}\left[\frac{d^{2}}{dr^{2}}\left(\sigma_{rr}\right)+\frac{1}{r}\left(\frac{d}{dr}\left(\sigma_{rr}\right)\right)\right]=\frac{E}{1-\mu^{2}}\left(\varepsilon_{rr}+\mu \varepsilon_{\theta \theta }\right)$$

(30) $$\sigma_{\theta \theta }-{\left(e_{0}a\right)}^{2}\left[\frac{d^{2}}{dr^{2}}\left(\sigma_{\theta \theta }\right)+\frac{1}{r}\left(\frac{d}{dr}\left(\sigma_{\theta \theta }\right)\right)\right]=\frac{E}{1-\mu^{2}}\left(\varepsilon_{\theta \theta }+\mu \varepsilon_{rr}\right)$$

Here, $\sigma_{rr}$ denotes the radial stress, $\sigma_{\theta \theta }$ denotes the circumferential stress, $\varepsilon_{rr}$ and $\varepsilon_{\theta \theta }$ denote the radial strain and the circumferential strain, respectively. Moreover, a refers to the internal characteristic length, whereas $e_{0}$ is the calibration constant’s value that depends on the material. The scaling factor that handles the small-scale effect is represented by $e_{0}a$, which is the product as a whole. We can obtain the sixth-order governing equation for the displacement in the non-local circular plate model by entering the values of $M_{rr}$, $M_{\theta \theta }$, and $Q_{r}$, into Equations (27) and (28), giving us the following results:

(31) $$\begin{array}{l} -{\left(e_{0}a\right)}^{2}\left[\begin{array}{l} r^{5}W_{n}^{VI}+7r^{4}W_{n}^{V}+\left(3+2\mu \right)r^{3}W_{n}^{IV}-\left(2+4\mu \right)\\ r^{2}W_{n}^{\prime \prime \prime }-\left(3-6\mu \right)\left(rW_{n}^{\prime \prime }-W_{n}^{\prime }\right) \end{array}\right]\\ +W_{n}^{IV}+\frac{2}{r}W_{n}^{\prime \prime \prime }-\frac{1}{r^{2}}W_{n}^{\prime \prime }+\frac{1}{r^{3}}W_{n}^{\prime }\\ =\frac{{\left(e_{0}a\right)}^{4}}{D}\left(F_{e}^{IV}+\left(\frac{6}{r}\right)F_{e}^{\prime \prime \prime }+\left(\frac{3}{r^{2}}\right)F_{e}^{\prime \prime }-\left(\frac{3}{r}\right)F_{e}^{\prime }\right)\\ -\frac{{\left(e_{0}a\right)}^{2}}{D}\left(2F_{e}^{\prime \prime }+\left(\frac{6}{r}\right)F_{e}^{\prime }\right)+\frac{F_{e}}{D} \end{array}$$

Equation (31) behaves in a similar way to the classical (local) plate model, when the scaling factor $e_{0}a$ is considered as zero. Employing the variable transformation technique with $\eta =r^{2}/{\left(2e_{0}a\right)}^{2}$ and disregarding the terms that lead to singularity when $e_{0}a\to 0$, the solution of Equation (31) is obtained as follows:

(32) $$W_{n}=C_{6}+\left(\frac{c_{2}}{{\left(2e_{0}a\right)}^{2}}\right)r^{2}+\left(\frac{F_{0}}{8\left(7+\nu \right)D}\right)r^{4}$$

Using boundary conditions, the deflection in the non-local plate theory is written as follows:

(33) $$W_{n}=\frac{P_{dc}{\left(a_{m}^{2}-r^{2}\right)}^{2}}{8\left(\nu +7\right)D_{0}}$$

Now, considering the solution of Ψ, we use $W=W_{n}$. In the non-local plate model, W of Equation (12) is given by $W_{n}$ of Equation (33). Using a similar type of formula, as discussed in detail in the plate model, the time-independent pressure profile in the non-local plate model is derived from $W_{n}^{\prime }=W_{n}\left(r=a_{m}\right)$ as follows:

(34) $$\begin{array}{l} \Psi \left(R\right)=\frac{32W_{n}^{\prime }}{j\sigma \left(\sqrt{-j\sigma }\right)J_{1}\left(\sqrt{-j\sigma }\right)}J_{0}\left(R\sqrt{-j\sigma }\right)-\\ W_{n}^{\prime }\left[\frac{64-\sigma^{2}{\left(R-1\right)}^{2}{\left(R+1\right)}^{2}+\left(16R^{2}-8\right)j\sigma }{\sigma^{2}}\right] \end{array}$$

## 3. Results and Discussion

Apart from using the membrane model and two types of plate models, the transducer considered in the present study has also been simulated with COMSOL (FEM model). The electromechanical physics is applied on the device to study the characteristics of the diaphragm for variable thicknesses and radii during FEM simulation. The natural frequency of Si_3_N_4_ CMUT, reported in [12] theoretically, is 87.148 KHz with a membrane thickness of 10 μm and membrane radius of 750 μm. The simulation in COMSOL for the same dimension of CMUT bearing vented vias exhibits an excellent agreement giving the frequency of 94.28 KHz, while the experimentally obtained frequency is 106 KHz [25] as discussed in our last work [45]. Badi et al. [46] have fabricated and verified the lamb wave micromachined capacitive transducers consisting of a series of rectangular membranes of silicon nitride with 1 cm in length and 100 μm in width.

In Table 1, the dimension of the device used during FEM simulation is presented. The different physical properties of different materials are acquired from the standard library of COMSOL. The values of some important parameters are enlisted in Table 2.

**Figure 2:**
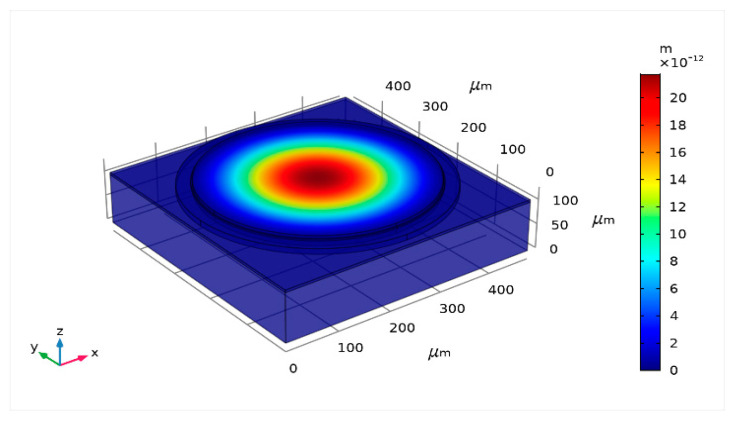
Displacement profile (a_m_ = 200 µm, t_m_ = 4 µm, t_g_ = 11.2 µm, V_dc_ = 40 V).

To understand the efficacy of a theoretical model in determining the displacement profile of the CMUT, a wide range of diaphragm radii (100 μm to 900 μm) and thicknesses (0.5 μm to 20 μm) are considered in the present study, and the results are compared with the COMSOL outputs (Figure 2) employing various statistical measures. All the calculations are carried out at the applied DC bias of 40 V. The high dimension of the diaphragm and high electrostatic load does not cause the compromise of aging of the device.

Absolute *QD* is a good measure to comprehend the performance of a model. Here, *QD* (square of the difference of displacement of the model and FEM) has been calculated at the membrane center (r = 0). We have considered four regions based on CMUT dimension to analyze the *QD* contour plots.

**Figure 3:**
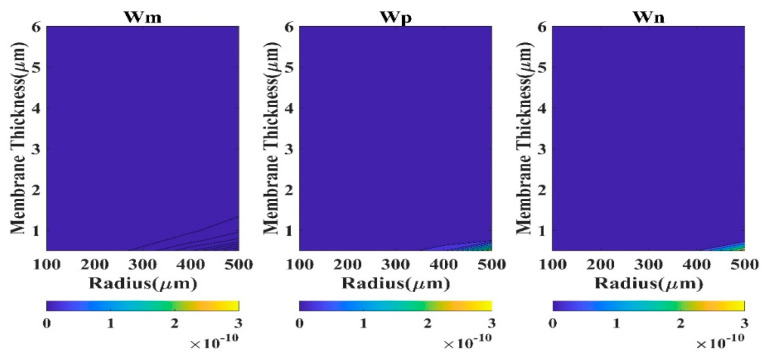
Contour plots of absolute *QD* in region R-I.

**Figure 4:**
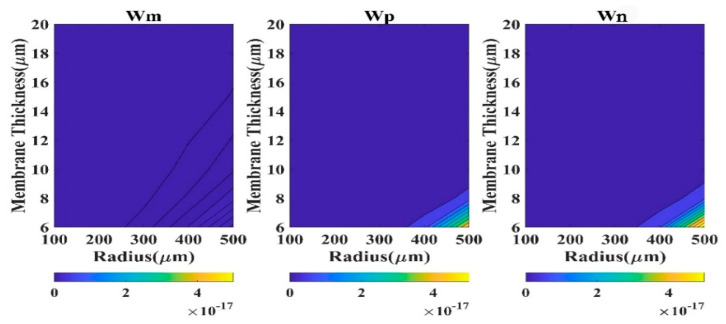
Contour plots of absolute *QD* in region R-II.

**Figure 5:**
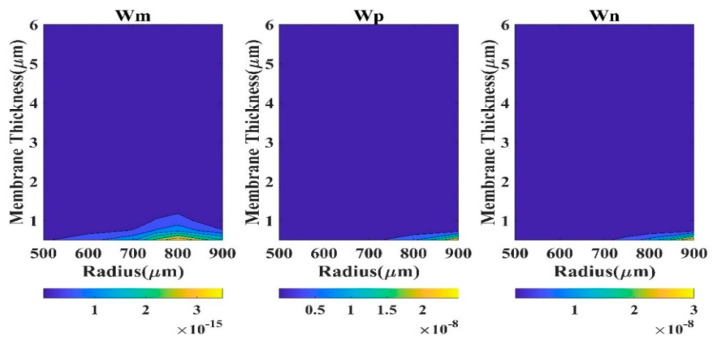
Contour plots of absolute *QD* in region R-III.

**Figure 6:**
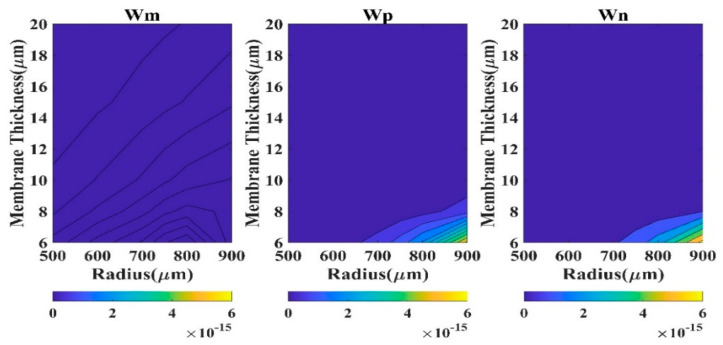
Contour plots of absolute *QD* in region R-IV.

The region R-I designates 0.5 µm ≤ t_m_ ≤ 6 µm and 100 µm ≤ a_m_ ≤ 500 µm. The second region R-II typifies 6 µm < t_m_ ≤ 20 µm and 100 µm ≤ a_m_ ≤ 500 µm, while region R-III represents 0.5 µm ≤ t_m_ ≤ 6 µm and 500 µm < a_m_ ≤ 900 µm. The fourth region R-IV exhibits the CMUT dimensions 6 µm < t_m_ ≤ 20 µm and 500 µm < a_m_ ≤ 900 µm. The contour plots of QD in various regions are depicted in Figure 3, Figure 4, Figure 5 and Figure 6 for the membrane model, plate model, and non-local plate model. All three models performed very well at R-II region, which can be realized from the very small magnitude of absolute quadratic deviation (QD). Though, in this small radius-large thickness zone, the membrane model is superior to the plate model in the very small subregion expressed by a_m_ > 450 µm and t_m_ < 7 µm. In R-IV region, both membrane and plate models perform quite well (QD). 

Moreover, the membrane model presented herein is superior to the plate model for large radius (a_m_ > 800 µm) and small thickness (t_m_ < 8 µm) zone. In region R-III, the performance of the plate model is very poor (QD), although the membrane model is capable of producing satisfactory outputs (QD).

In region R-I, both membrane and plate models’ outputs have been degraded (*QD* $<3\times 10^{-10}$). Therefore, for substantive membrane thickness (t_m_ > 2 µm), the performances of all the three models are acceptable. For small diaphragm thickness, especially when a_m_/t_m_ > 1000, CMUT is well expressed by the membrane model compared to the plate models.

**Figure 7:**
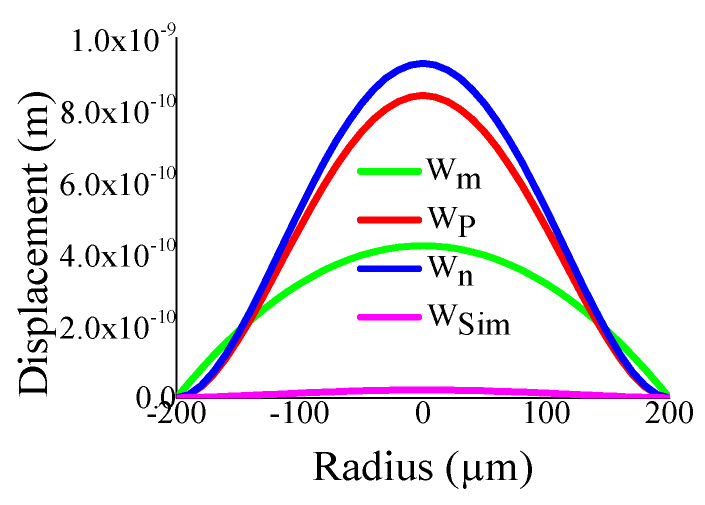
Displacement with radial position r for t_g_ = 11.2 µm, V_dc_ = 40 V, a_m_ = 200 µm, t_m_ = 4 µm.

**Figure 8:**
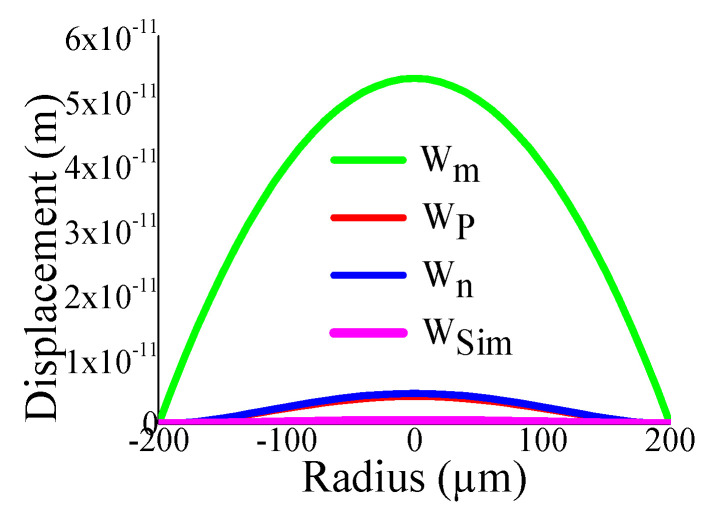
Displacement with radial position r for t_g_ = 11.2 µm, V_dc_ = 40 V, a_m_ = 200 µm, t_m_ = 20 µm.

**Figure 9:**
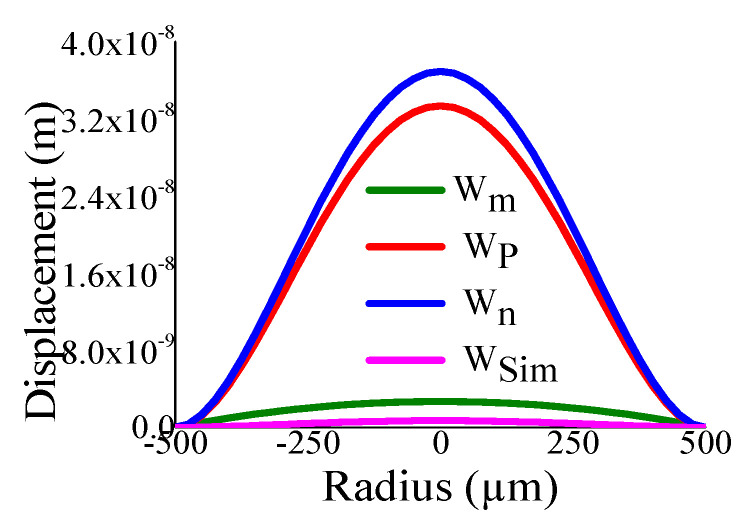
Displacement with radial position r for t_g_ = 11.2 µm, V_dc_ = 40 V, a_m_ = 500 µm, t_m_ = 4 µm.

**Figure 10:**
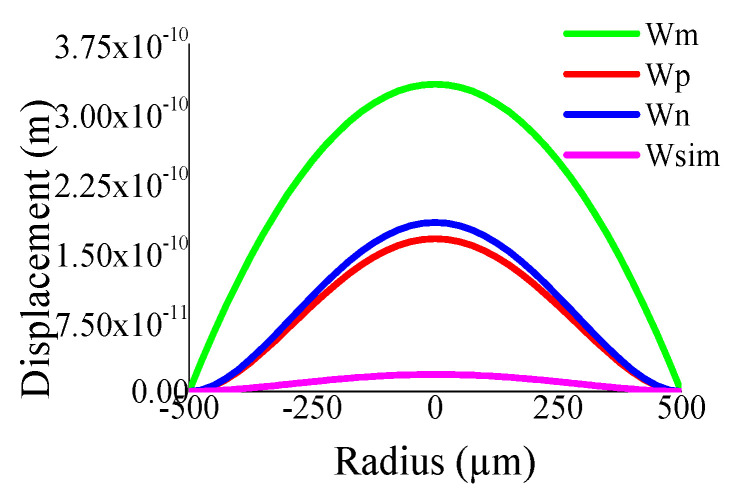
Displacement with radial position r for t_g_ = 11.2 µm, V_dc_ = 40 V, a_m_ = 500 µm, t_m_ = 20 µm.

Some of the displacement profiles have been shown in (Figure 7, Figure 8, Figure 9 and Figure 10), where *W_m_*, *W_p_*, and *W_n_* represent the outputs of the membrane, plate, and non-local plate models, while *W_sim_* presents the COMSOL outcome for the applied DC bias V_dc_ *=* 40 V and CMUT air gap thickness t_g_ *=* 11.2 µm. It is interesting to observe that the performance of plate models is better than the membrane model for t_m_ > 15 µm, while the membrane model is preferred at t_m_ > 5 µm.

**Figure 11:**
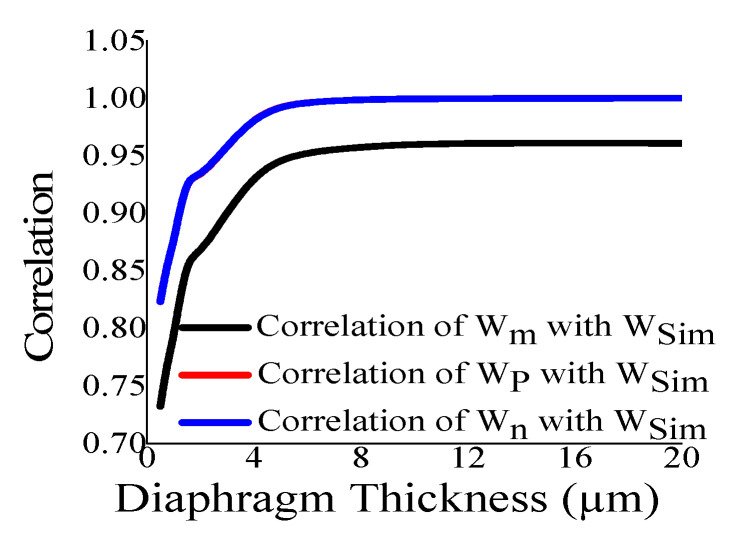
Correlation of the displacement of analytical model and the simulation at the center of the diaphragm with varying t_m_.

**Figure 12:**
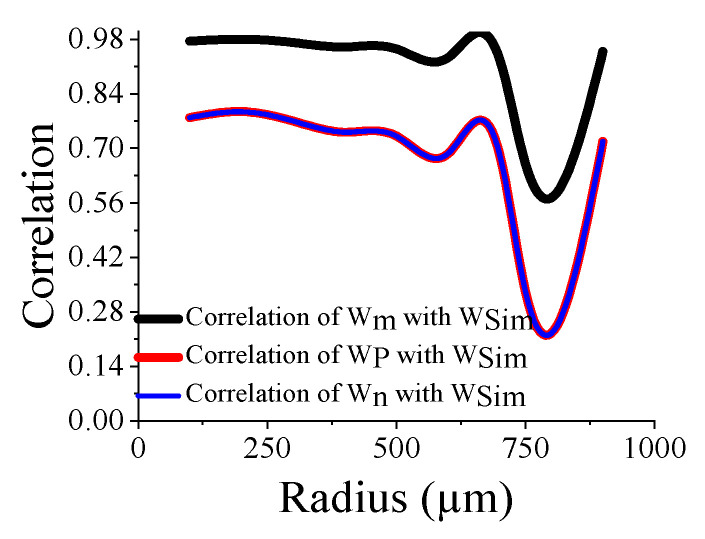
Correlation of the displacement of analytical model and the simulation at the center of the diaphragm with varying a_m_.

**Figure 13:**
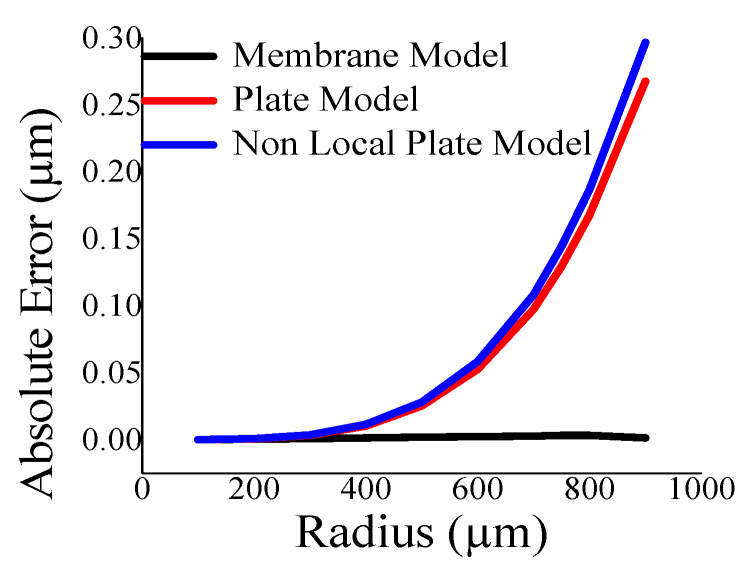
Absolute error of displacement at the center of diaphragm with varying a_m_ at t_m_ = 4 µm.

**Figure 14:**
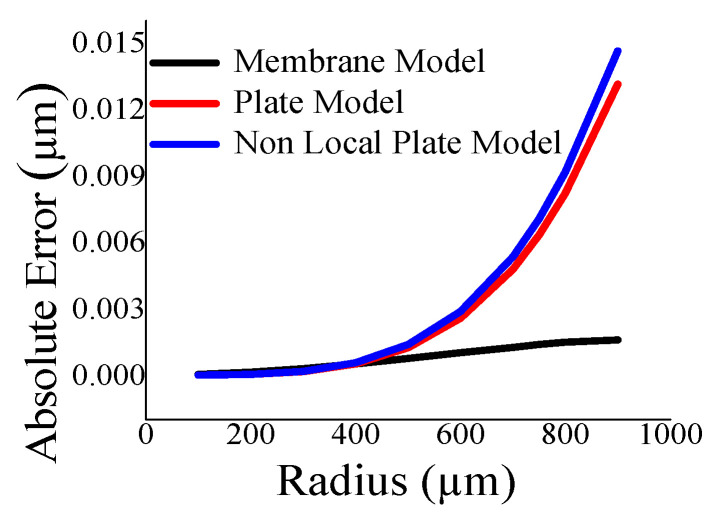
Absolute error of displacement at the center of diaphragm with varying a_m_ at t_m_ = 10 µm.

**Figure 15:**
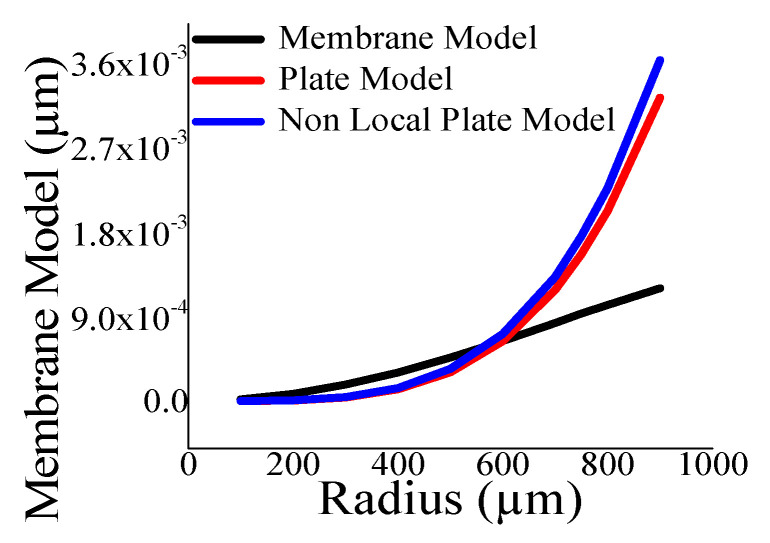
Absolute error of displacement at the center of diaphragm with varying a_m_ at t_m_ = 15 µm.

**Figure 16:**
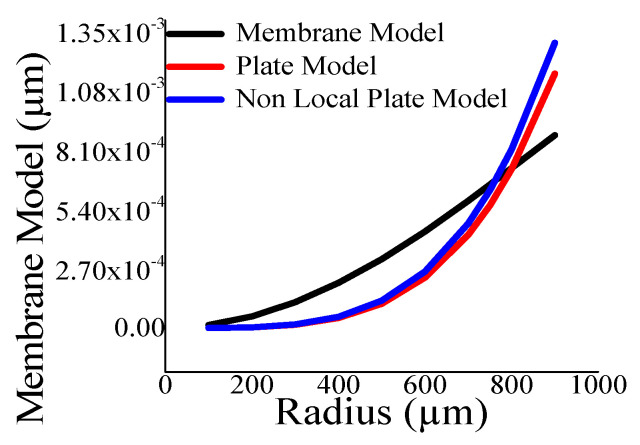
Absolute error of displacement at the center of diaphragm with varying a_m_ at t_m_ = 20 µm.

**Figure 17:**
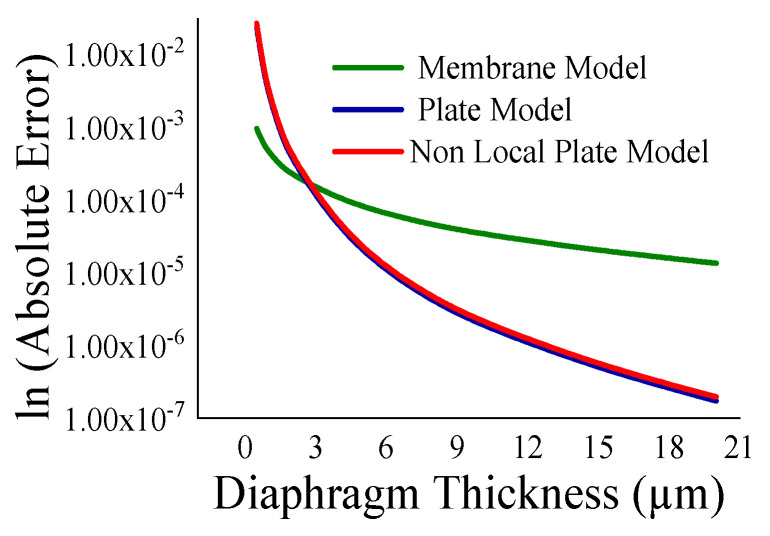
Logarithm absolute error of displacement at the centre of diaphragm with varying t_m_ for a_m_ = 100 µm.

**Figure 18:**
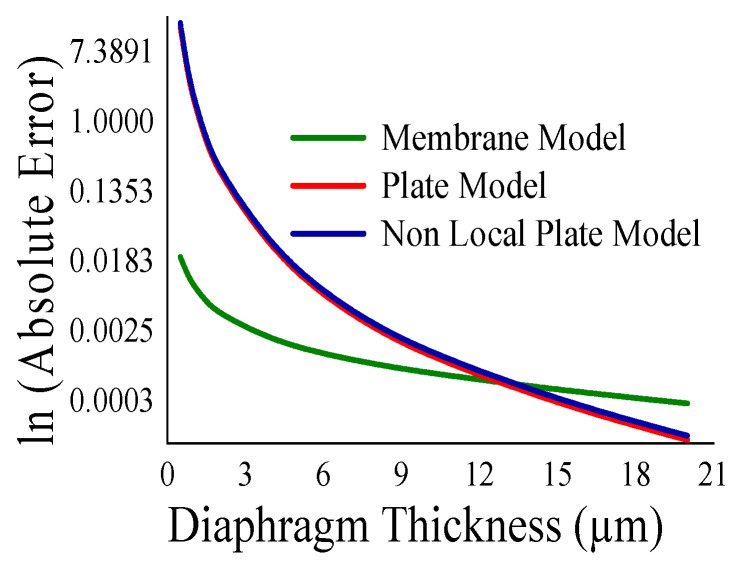
Logarithm absolute error of displacement at the centre of diaphragm with varying t_m_ for a_m_ = 500 µm.

**Figure 19:**
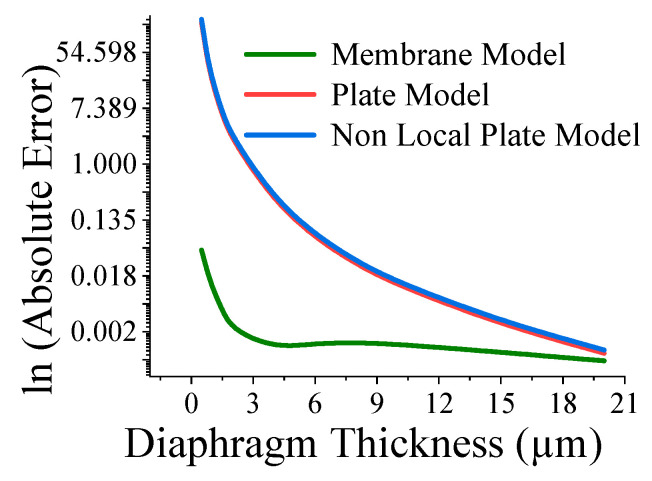
Logarithm absolute error of displacement at the centre of diaphragm with varying t_m_ for a_m_ = 900 µm.

Here, the closeness of the displacement predicted by the mathematical model and the simulation has also been studied. It is reasonable for t_m_ > 4 µm (Figure 11) and a_m_ < 700 µm (Figure 12). The variations of correlation of *W_n_* and *W_p_* in Figure 11 and Figure 12 are identical, and thus overlapped. The variations of absolute error of displacement at the center of the diaphragm with varying radii are depicted in (Figure 13, Figure 14, Figure 15 and Figure 16) for various membrane thicknesses. Similarly, the variations of the logarithm of absolute error with various thicknesses are represented in (Figure 17, Figure 18 and Figure 19) for a_m_ as equal to 100 µm, 500 µm, and 900 µm. This absolute deviation analysis has also established that when the diaphragm thickness is small (t_m_ < 10 µm) and radius is large (a_m_ > 500 µm), the outputs of plate models significantly deviate with respect to the COMSOL outputs, though the membrane model performs well.

**Figure 20:**
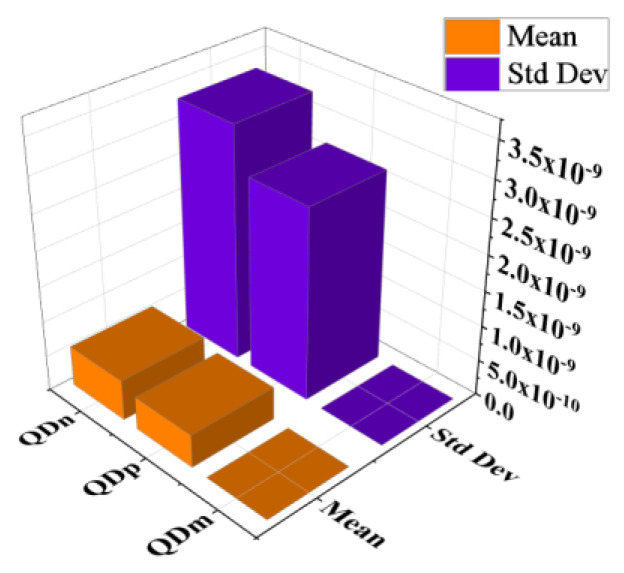
Mean and standard deviation of absolute *QD* for models in the range 100–900 μm for a_m_ and 0.5–20 μm for t_m_.

Figure 17, Figure 18 and Figure 19 show that the variations of absolute error of the non-local plate model exactly follow the pattern of the plate model. Therefore, it is difficult to distinguish them in graphs. With an increase in the membrane thickness, the performances of the plate models improve substantially, and for CMUT dimension of small radius, they become superior to the membrane model. To quantify, comprehend, and summarize the performance of these models region-wise, overall, the mean ($\bar{QD}$) and standard deviation of *QD* ($\sigma_{QD}$) is estimated as presented in Table 3. In each and every case, plate models are inferior to the membrane model, while the general plate model is better than the non-local plate version. The summary of the overall performance of the models is exhibited in Figure 20.

**Figure 21:**
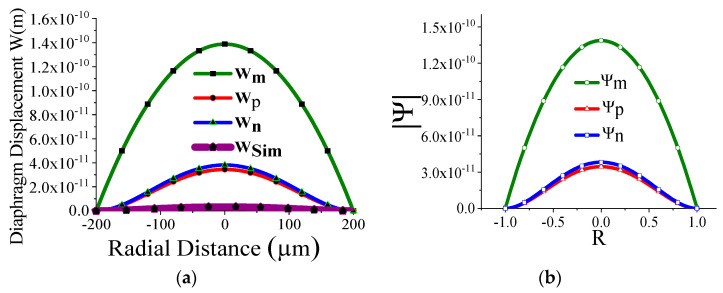
Displacement profile W(r) (**a**) and pressure profile Ψ(R) (**b**) for a_m_ = 200 μm, t_m_ = 10 µm, t_g_ = 11.2 µm at V_dc_ = 40 V.

**Figure 22:**
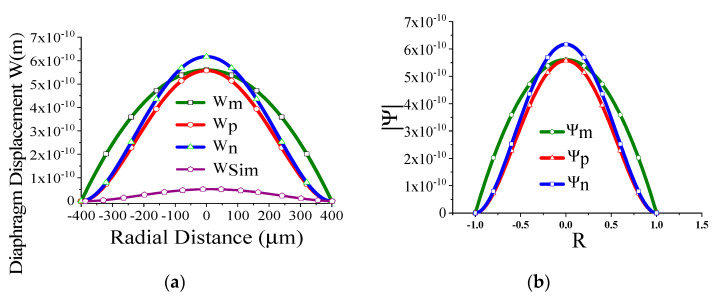
Displacement profile W(r) (**a**) and pressure profile Ψ(R) (**b**) for a_m_ = 400 μm, t_m_ = 10 µm, t_g_ = 11.2 µm at V_dc_ = 40 V.

**Figure 23:**
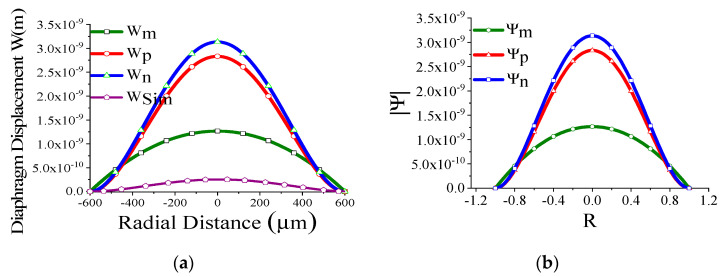
Displacement profile W(r) (**a**) and pressure profile Ψ(R) (**b**) for a_m_ = 600 μm, t_m_ = 10 µm, t_g_ = 11.2 µm at V_dc_ = 40 V.

**Figure 24:**
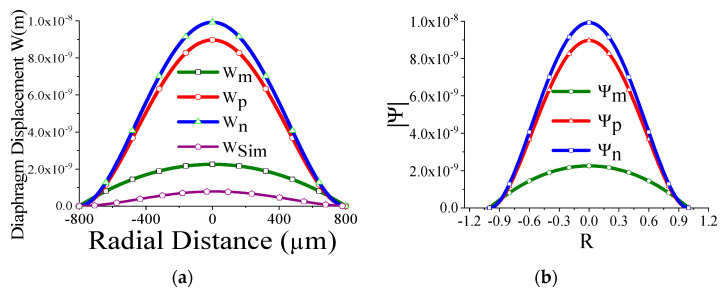
Displacement profile W(r) (**a**) and pressure profile Ψ(R) (**b**) for a_m_ = 800 μm, t_m_ = 10 µm, t_g_ = 11.2 µm at V_dc_ = 40 V.

We have thoroughly investigated the distribution of pressure in three different theoretical models and observed that the pressure profile follows almost the displacement profile, since no damping is expected in an air-filled (at atmospheric pressure) sealed CMUT. As these theoretical models perform very well in regions II and IV, we have represented both the displacement profile and pressure profile in these regions for the radius of the membrane of 200 µm, 400 µm, 600 µm, and 800 µm in Figure 21, Figure 22, Figure 23 and Figure 24 for t_m_ = 10 µm, t_g_ = 11.2 µm at V_dc_ = 40 V. As the radius increases, the overall magnitude of pressure also increases. It is interesting to note that the pressure profile decreases exponentially with an increase in t_m_, and thus after a certain limit, it saturates.

**Figure 25:**
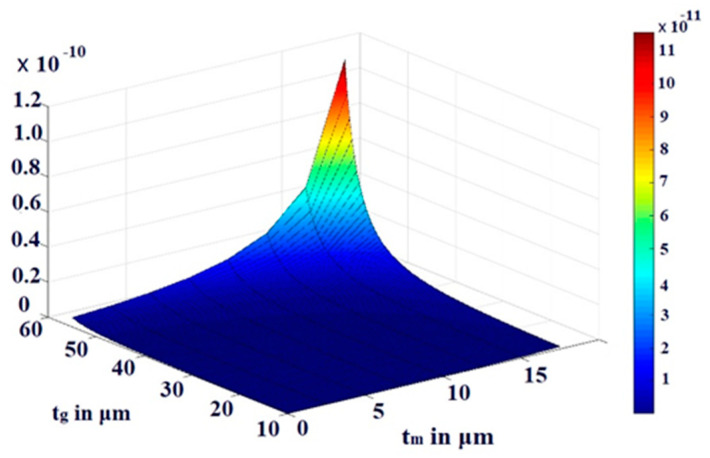
The 3D plot of pressure profile Ψ with variation of t_m_ and t_g_ at R = 0 for a_m_ = 750 μm and V_dc_ = 40 V in membrane model.

**Figure 26:**
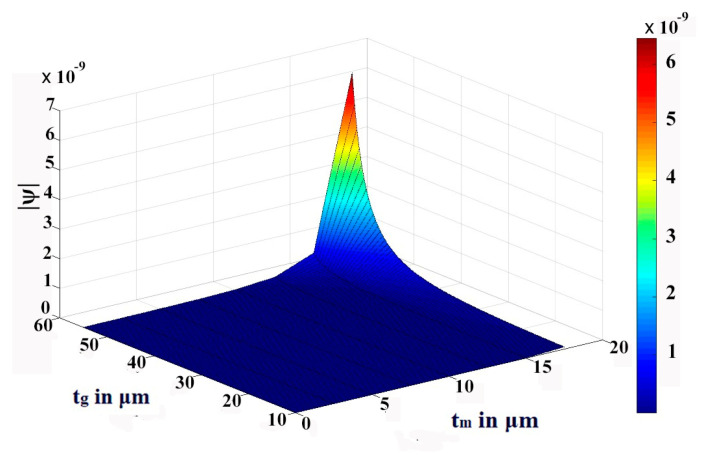
The 3D plot of pressure profile Ψ with variation of t_m_ and t_g_ at R = 0 for a_m_ = 750 μm and V_dc_ = 40 V in plate model.

**Figure 27:**
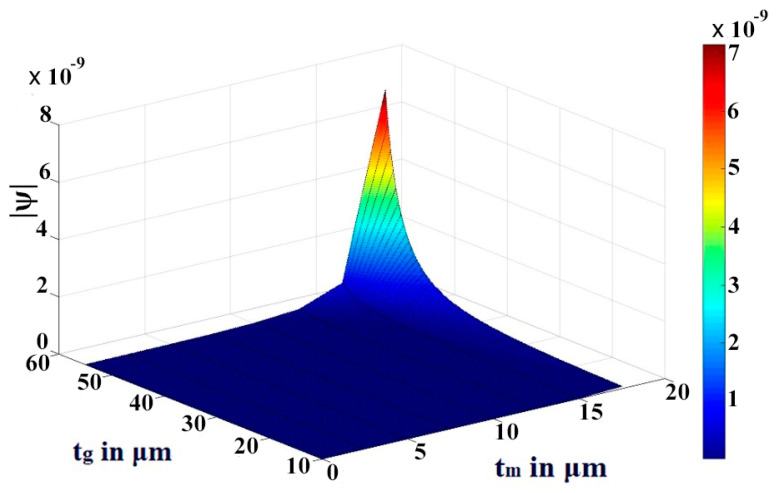
The 3D plot of pressure profile Ψ with variation of t_m_ and t_g_ at R = 0 for a_m_ = 750 μm and V_dc_ = 40 V in non-local plate model.

The variations are more prominent in plate models compared to the membrane model. A similar type of observation is also found in the case of variations of pressure with t_g_. These results of variations of pressure profile Ψ with t_m_ and t_g_ are depicted in Figure 25, Figure 26 and Figure 27 through the 3D plots for the membrane model, plate model, and non-local plate model, respectively.

**Figure 28:**
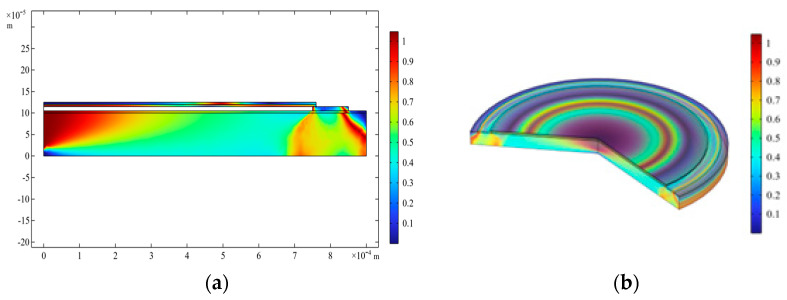
FEM simulation of Lode angle for a_m_ = 750 μm and V_dc_ = 40 V in (**a**) 2D and (**b**) 3D.

**Figure 29:**
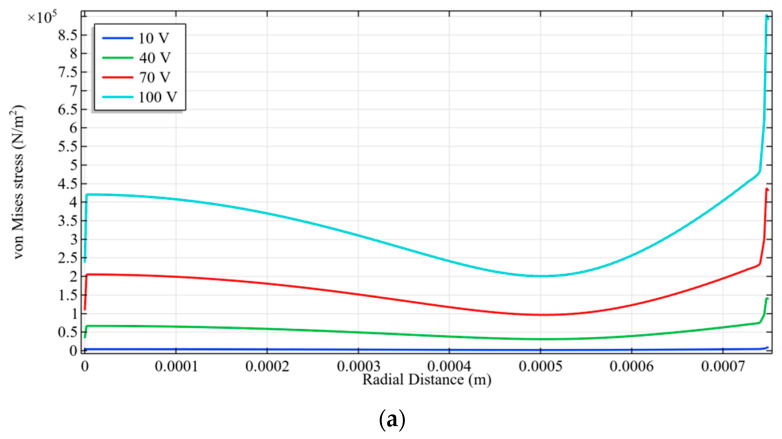

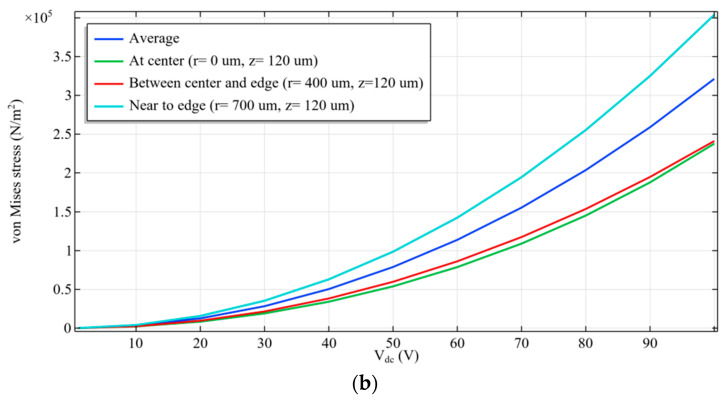
Variation of simulated von Mises stress (**a**) with radial distance for a_m_ = 750 μm and V_dc_ = 40 V (**b**) stress with DC bias for a_m_ = 750 μm.

To optimize the aging and sensitivity of detection of pressure profile of the proposed device, a thorough investigation of lode angle (Figure 28) and Von Mises stress analysis (Figure 29) has been carried out.

## 4. Conclusions

In accordance with the identical geometrical shape of the manufactured structure, this model predicts a resonance frequency of 94.28 KHz, which is quite near to the outcome of the experiment (106 KHz). In the present study, the time-independent pressure profile of air trapped circular Si_3_N_4_ CMUT being clamped on both sides, has been studied analytically in the framework of the plate model and the membrane model. In all these models, the pressure profile follows almost the displacement profile. As the radius of the diaphragm increases, the overall magnitude of pressure also increases. On the other hand, the pressure profile decreases exponentially with increases in both membrane thickness and air gap thickness. Moreover, the displacement profiles are studied in detail considering the range of diaphragm radii 100–900 µm and diaphragm thicknesses 0.5–20 µm. A comparison of mathematical results and COMSOL simulation has been carried out to reach an idea of the preferential choice of a theoretical model for a given CMUT dimension. For this, we have proposed the systematic study of contour plots of absolute quadratic deviation and many other statistical measures. These statistical analyses indicate that the displacement outputs are acceptable for the diaphragm thickness of more than 2 µm. In this dimension, the membrane model is preferred in the case of the thickness of diaphragm of less than 5 µm, and plate models are ideal for the diaphragm thickness of more than 15 µm. In the regime, t_m_ > 4 µm and a_m_ < 700 µm, the correlation of displacement profile prediction in these models is agreeable. Interestingly, while the plate models perform worst in the large radius-small thickness zone where a_m_/t_m_ > 1000, the membrane model predicts the displacement profile quite well. The examination and analysis of absolute quadratic deviation and absolute deviation of displacement profile reveal that the membrane model outputs are more satisfactory than those of plate models, while the general plate model outputs are better than the non-local plate version in the context of dimension range considered in the present study.

## Figures and Tables

**Table 1:** DEVICE DIMENSION.

Region	Dimension in μm
The thickness of the substrate	100
The thickness of the bottom electrode	5
The thickness of the air cavity	11.2
The thickness of the diaphragm	0.5, 0.75, 1.0, 1.5, 2.0, 4.0, 6.0, 8.0, 10.0, 15.0, 20.0
The radius of the diaphragm	50, 100, 200, 300, 400, 500, 600, 700, 750, 800, 900

**Table 2:** SIMULATION PARAMETERS.

Material	Property Name	Value
Silicon Nitride	Relative permittivity	9.7
	Density	3100 kg/m^3^
	Young’s modulus	250 × 10^9^ Pa
	Poisson’s ratio	0.23
	Thermal conductivity	20 W/m·K
	Coefficient of thermal expansion	2.3 × 10^−6^ K^−1^
Silicon	Coefficient of thermal expansion	2.6 × 10^−6^ K^−1^
	Heat capacity at constant pressure	700 J/kg·K
	Relative permittivity	11.7
	Density	2329 kg/m^3^
	Thermal conductivity	130 W/m·K
	Young’s modulus	170 × 10^9^ Pa
	Poisson’s ratio	0.28
Air	Relative permittivity	1.00

**Table 3:** Mean and standard deviation of absolute quadratic deviation.

Region	Model	$\bar{QD}$	$\sigma_{QD}$
I	Membrane Model	$3.54\times 10^{-17}$	$8.34\times 10^{-17}$
	Plate Model	$8.63\times 10^{-12}$	$3.94\times 10^{-11}$
	Non-local Plate Model	$1.06\times 10^{-11}$	$4.86\times 10^{-11}$
II	Membrane Model	$2.34\times 10^{-19}$	$3.94\times 10^{-19}$
	Plate Model	$2.61\times 10^{-18}$	$9.38\times 10^{-18}$
	Non-local Plate Model	$3.23\times 10^{-18}$	$1.16\times 10^{-17}$
III	Membrane Model	$4.92\times 10^{-16}$	$8.14\times 10^{-16}$
	Plate Model	$1.43\times 10^{-9}$	$4.64\times 10^{-9}$
	Non-local Plate Model	$1.75\times 10^{-9}$	$5.68\times 10^{-9}$
IV	Membrane Model	$1.69\times 10^{-18}$	$1.30\times 10^{-18}$
	Plate Model	$3.72\times 10^{-16}$	$1.01\times 10^{-15}$
	Non-local Plate Model	$4.60\times 10^{-16}$	$1.26\times 10^{-15}$
All	Membrane Model	$1.66\times 10^{-16}$	$5.15\times 10^{-16}$
	Plate Model	$4.67\times 10^{-10}$	$2.71\times 10^{-9}$
	Non-local Plate Model	$5.72\times 10^{-10}$	$3.32\times 10^{-9}$

## Data Availability

All data that support the findings of this study are included within the article.
